# The innate interferon gamma response of BALB/c and C57BL/6 mice to *in vitro Burkholderia pseudomallei *infection

**DOI:** 10.1186/1471-2172-7-19

**Published:** 2006-08-18

**Authors:** Ghee Chong Koo, Yunn-Hwen Gan

**Affiliations:** 1 Department of Biochemistry, Yong Loo Lin School of Medicine, National University of Singapore, MD7, 8 Medical Drive, Singapore 117597, Republic of Singapore; 2 Immunology Program, National University of Singapore, OLS Satellite Laboratory, DMERI Building, 27 Medical Drive, Singapore 117510, Republic of Singapore

## Abstract

**Background:**

*Burkholderia pseudomallei *is the causative agent for melioidosis. For many bacterial infections, cytokine dysregulation is one of the contributing factors to the severe clinical outcomes in the susceptible hosts. The C57BL/6 and BALB/c mice have been established as a differential model of susceptibility in murine melioidosis. In this study, we compared the innate IFN-γ response to *B. pseudomallei *between the C57BL/6 and BALB/c splenocytes and characterized the hyperproduction of IFN-γ in the relatively susceptible BALB/c mice *in vitro*.

**Results:**

Naïve BALB/c splenocytes were found to produce more IFN-γ in response to live bacterial infection compared to C57BL/6 splenocytes. Natural killer cells were found to be the major producers of IFN-γ, while T cells and Gr-1^intermediate ^cells also contributed to the IFN-γ response. Although anti-Gr-1 depletion substantially reduced the IFN-γ response, this was not due to the contribution of Gr-1^high^, Ly-6G expressing neutrophils. We found no differences in the cell types making IFN-γ between BALB/c and C57BL/6 splenocytes. Although IL-12 is essential for the IFN-γ response, BALB/c and C57BL/6 splenocytes made similar amounts of IL-12 after infection. However, BALB/c splenocytes produced higher proinflammatory cytokines such as IL-1β, TNF-α, IL-6, IL-18 than C57BL/6 splenocytes after infection with *B. pseudomallei*.

**Conclusion:**

Higher percentages of Gr-1 expressing NK and T cells, poorer ability in controlling bacteria growth, and higher IL-18 could be the factors contributing to IFN-γ hyperproduction in BALB/c mice.

## Background

*Burkholderia pseudomallei *is the causative agent for melioidosis, an infectious disease endemic in South-east Asia and northern Australia [1,2]. It has also been increasingly reported in other tropical and subtropical regions [3]. The bacillus is a facultative intracellular microbe and can invade and replicate in many different organs. Infection can result in a wide spectrum of clinical outcomes, ranging from an asymptomatic state, benign pulmonitis, acute or chronic pneumonia, and to fulminant septicemias [4]. Furthermore, even after the apparent resolution of acute symptoms, the infection can persist for decades as a chronic and latent condition where relapse is possible [5]. Despite appropriate antibiotic treatment, severe melioidosis with acute septicemia is associated with a high mortality rate [6].

In severe melioidosis, patients exhibit elevated serum levels of proinflammatory cytokines such as TNF-α [7], IFN-γ [8] and IFN-γ induced chemokines IP-10 and MIG [9]. Murine models of acute melioidosis mimic human pathology. mRNA for proinflammatory cytokines such as TNF-α, IFN-γ and IL-6 were detected earlier and in more abundance in the organs of BALB/c mice with acute disease than the more resistant C57BL/6 mice when they were infected intravenously [10]. We had previously established an intranasal murine model where BALB/c mice were susceptible while C57BL/6 mice were relatively more resistant to disease. We found high transient levels of IFN-γ both locally and systemically in susceptible mice, which exhibit acute disease followed by death within a week after infection [11]. The high levels of IFN-γ correlated with high bacterial loads in the organs [11]. In another study, administering CpG DNA prior to bacterial challenge could attenuate hyperproduction of IFN-γ in serum of BALB/c mice while lowering the bacterial load in the blood at the same time [12]. So although IFN-γ was shown to be critical in host survival in the first 24 h after infection as neutralizing antibodies against IFN-γ lowered the LD_50 _by approximately 100, 000 fold [13], hyperproduction could contribute to immune pathology and severe disease. We are interested in comparing the innate IFN-γ response to *B. pseudomallei *between C57BL/6 and BALB/c mice, and in characterizing the hyperproduction of IFN-γ in BALB/c through the *in vitro *stimulation of naïve splenocytes with heat-killed or live bacteria. We found that naïve BALB/c splenocytes consistently produce more IFN-γ in response to live bacterial infection compared to C57BL/6 splenocytes. Through various comparisons between BALB/c and C57BL/6 splenocytes, factors which could contribute to the hyperproduction of IFN-γ in BALB/c splenocytes are discussed.

## Results

### BALB/c and C57BL/6 splenocytes produce IFN-γ when stimulated with *B. pseudomallei*

**Figure 1:**
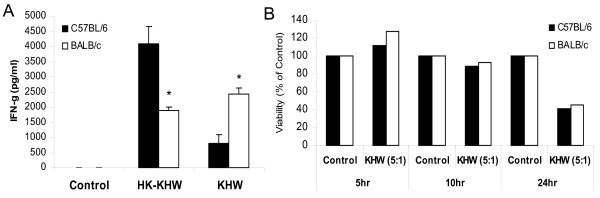
**Differential IFN-γ response of BALB/c and C57BL/6 splenocytes to heat-killed and live *B. pseudomallei *stimulation**. Naïve splenocytes were stimulated with heat-killed *B. pseudomallei *strain KHW (HK-KHW) or live *B. pseudomallei *strain KHW. The ratio for HK-KHW to cells is 5:1, whereas the MOI with live bacteria is 5:1. Supernatants were harvested at 24 h for determination of IFN-γ by ELISA (A). Cell viability of *B. pseudomallei *infected splenocytes was examined by the XTT assay at 5 h, 10 h and 24 h post-infection (B). Asterisks * represent statistical significance (p < 0.05) as determined by the Student t test. All experiments had been repeated at least 3 times.

It had been previously reported that splenocytes from naïve animals could produce IFN-γ in response to gamma irradiated *B. pseudomallei *[14]. In order to further characterize the IFN-γ response of BALB/c and C57BL/6 to *B. pseudomallei*, we determine if naïve splenocytes from these mice could produce IFN-γ when infected with bacteria *in vitro*. Under optimal bacteria to cell ratio, we found that naïve splenocytes produced high amounts of IFN-γ with heat-killed and live *B. pseudomallei*, detectable by 12 h (data not shown) and at 24 h (Fig. 1A). Although there were individual variances from mouse to mouse, naïve BALB/c splenocytes consistently produced more IFN-γ in response to live *B. pseudomallei *compared to those from C57BL/6 mice whereas naïve C57BL/6 splenocytes produced significantly higher IFN-γ than BALB/c splenocytes when treated with heat-killed bacteria. Thus, the pattern of IFN-γ production to live bacteria mimics the infection *in vivo*. Since higher IFN-γ production in naïve BALB/c splenocytes upon live bacterial infection *in vitro *could not have resulted from increased infiltration of cell-types from elsewhere, we tested the possibility that naïve BALB/c splenocytes produce more IFN-γ in response to live bacteria due to a poorer ability to control bacterial replication, leading to a higher bacterial load. We found no difference in the total bacteria counts between BALB/c and C57BL/6 splenocytes at 4 h after infection in three separate experiments. The mean intracellular bacterial loads of splenocytes from three animals were higher in BALB/c (1.06 × 10^4 ^CFU) than C57BL/6 (7.77 × 10^3 ^CFU) at 4 h after infection, reaching a statistical significant value with p < 0.05, although the difference did not reach statistical significance in one out of the three separate experiments. Each experiment on either total or intracellular bacterial counts involves splenocytes from six animals, three from each inbred strain. Intracellular bacterial loads at later time-points were inconsistent and unreliable probably due to increasing cell death. Cell viability at various time points after infection was also determined to confirm that the differential response was not contributed by difference in cell viability after infection. Infection of cells did not affect the viability at 5 and 10 h post infection, but showed a > 50% reduction at 24 h post-infection, as compared to the uninfected control splenocytes (Fig. 1B). However, no differences in the percentages of cell viability between infected BALB/c and C57BL/6 splenocytes were detected even at 24 h post-infection.

### Comparison of cytokine profile between BALB/c and C57BL/6 splenocytes

**Figure 2:**
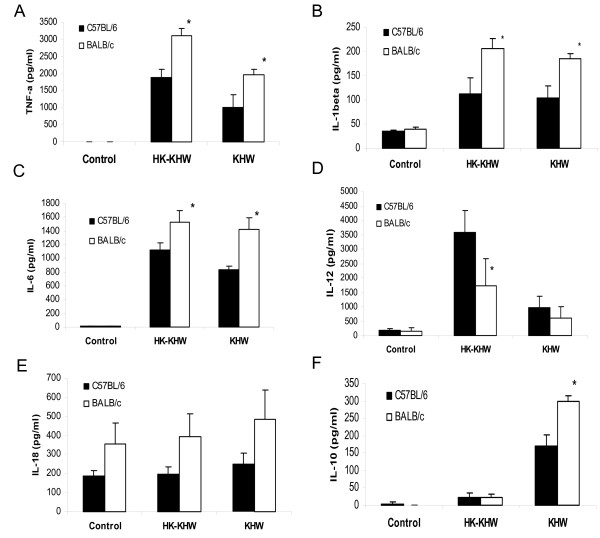
**Cytokine responses of BALB/c and C57BL/6 splenocytes to *B. pseudomallei *infection**. Splenocytes were infected with live *B. pseudomallei *strain KHW at MOI of 5:1. Supernatants were harvested at 24 h post-infection for ELISA for (A) TNF-α, (B) IL-1β, (C) IL-6, (D) IL-12, (E) IL-18 and (F) IL-10. Asterisks * represent statistical significance (p < 0.05) as determined by the Student t test.

*B. pseudomallei *has been demonstrated to induce non-polarized Th1 or Th2 cytokine responses *in vivo *[10,11]. Since naïve BALB/c splenocytes showed a higher IFN-γ response compared to naïve C57BL/6 splenocytes when infected *in vitro *with *B. pseudomallei*, we determine if there are similar differences in other proinflammatory cytokines such as TNF-α, IL-1β, IL-6, IL-12, IL-18 and the anti-inflammatory cytokine IL-10. Our results show that *B. pseudomallei *induced significantly higher concentrations of proinflammatory cytokines including TNF-α (Fig. 2A), IL-1β (Fig. 2B) and IL-6 (Fig. 2C) at 24 h in naïve BALB/c than C57BL/6 splenocytes. There was no difference in IL-12 amounts in the infected splenocytes (Fig. 2D). We were unable to detect an increase in IL-18 upon bacterial stimulation due to a constitutively high background although BALB/c still made more IL-18 than C57BL/6 splenocytes (Fig. 2E). The anti-inflammatory cytokine IL-10 was detected after infection of splenocytes from both strains of mice, with BALB/c producing a significantly higher amount of IL-10 as compared to C57BL/6, although dead bacteria did not induce much IL-10 from both (Fig. 2F). In response to heat-killed bacteria, BALB/c splenocytes produced more IL-1β, TNF-α and IL-6, but significantly less IL-12 when compared to C57BL/6 splenocytes. Thus, it appears that the higher IFN-γ response seen in C57BL/6 to dead bacteria correlated with higher IL-12 levels.

### IL-12 and IL-18 are both required for optimal IFN-γ response but IL-10 has a suppressive effect

**Figure 3:**
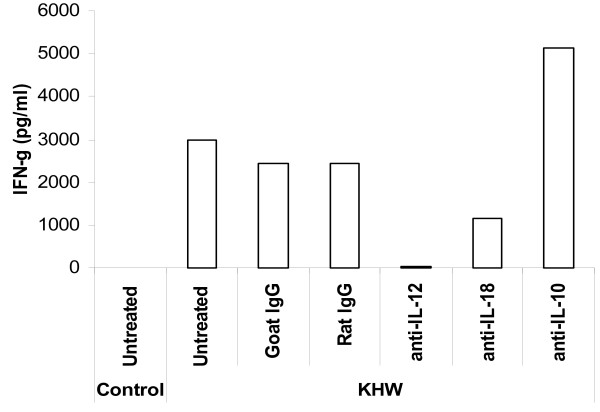
**The effects of cytokine neutralizing antibodies on the production of IFN-γ**. Splenocytes were harvested from BALB/c mice and preincubated with anti-IL-12 anti-IL-18, anti-IL-10 or their respective isotype control antibodies, followed by treatment with live *B. pseudomallei *strain KHW. Supernatants were harvested at 24 h for determination by ELISA. The experiment had been repeated 3 times with the same results.

The synergistic requirement of IL-12 and IL-18 for induction of IFN-γ is well-established [15]. To confirm the role of IL-12 and IL-18 in *B. pseudomallei *induced IFN-γ response, neutralizing antibodies against IL-12 and IL-18 were used. Addition of 1 μg/ml of neutralizing IL-12 antibodies could completely abrogate the production of IFN-γ by BALB/c splenocytes to live bacteria (Fig. 3). Neutralizing IL-18 antibodies partially lowered the amount of IFN-γ. The isotype control antibodies had no effect on IFN-γ. Furthermore, the dependence on IL-12 and IL-18 in the IFN-γ response was specific for bacterial stimulation, and not for PMA and ionomycin (data not shown). As splenocytes from both mouse strains make IL-10, which can be antagonistic to IFN-γ, we depleted IL-10 using neutralizing anti-IL-10 antibodies. Neutralizing anti-IL-10 increased the production of IFN-γ to more than 1.5 fold compared to the control. Similar results were observed when these neutralizing antibodies were used for infected C57BL/6 splenocytes, although the amount of IFN-γ induced by live *B. pseudomallei *was much lower (data not shown).

### Similar cell-types produce IFN-γ in response to bacteria in BALB/c and C57BL/6

**Figure 4:**
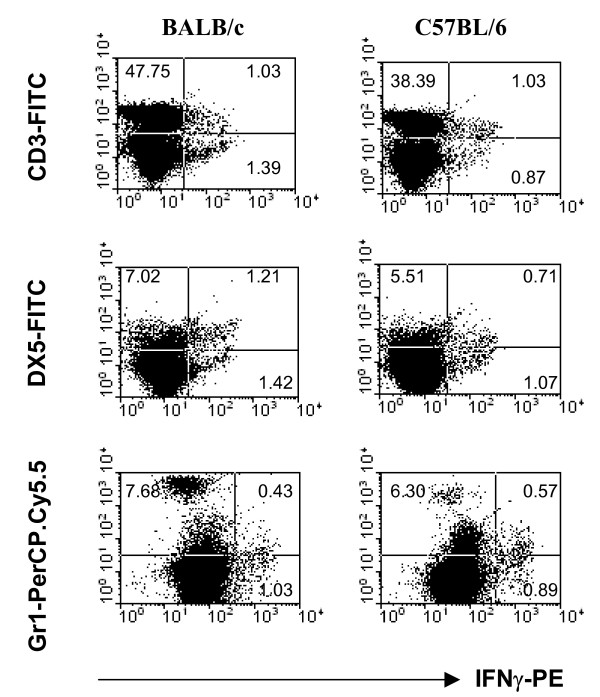
**IFN-γ production by BALB/c and C57BL/6 splenocytes as measured by the IFN-γ capture assay**. Splenocytes were infected with live *B. pseudomallei *strain KHW for 16 hours before staining was performed. Representative of two separate experiments is shown.

**Figure 5:**
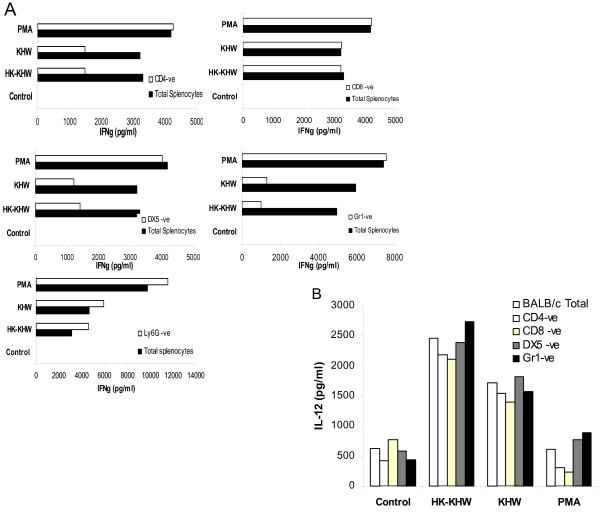
**The effect of cell subset depletion on the IFN-γ and IL-12 response**. Splenocytes from naïve BALB/c mice were subjected to CD4^+^, CD8^+^, DX5^+^, Gr-1^+ ^or Ly-6G^+ ^cell depletion by MACS and treated with heat-killed or live *B. pseudomallei *strain KHW. PMA and ionomycin stimulation served as positive controls, and the untreated total splenocytes served as negative controls. IFN-γ (A) and IL-12 (B) concentrations were determined by ELISA from supernatants harvested at 24 h after stimulation. Results shown are representative of at least 3 experiments.

To determine if the cell types producing IFN-γ vary between BALB/c and C57BL/6 splenocytes, we measured IFN-γ production by the IFN-γ capture assay using live bacteria as stimuli. In agreement with what others had found [16], our results show that T and NK cells were the major cells making IFN-γ (Fig. 4). The IFN-γ capture assay also reveals Gr-1^intermediate ^cells making IFN-γ. We also performed depletion of various cell types from bulk splenocytes. Only data for BALB/c mice are shown as results from both mouse strains are similar (Fig 5A). Percentage of depletion for Gr-1^high^, CD4^+^, CD8^+ ^and DX5^+ ^was at least > 85% as determined by flow cytometry. Depletion of B cells had no effect on IFN-γ production (data not shown). However, depletion of Gr-1^high ^cells, which depleted a significant proportion of Gr-1^intermediate ^cells as well, reduced IFN-γ production drastically both at 12 h (data not shown) and 24 h after stimulation (Fig. 5A). NK cells and CD4 T cells also contributed significantly to IFN-γ production. As Gr-1^high ^cells were generally associated with granulocytes such as neutrophils, although other cell types also expressed Gr-1 with varying intensity, we depleted the cultures with Ly-6G antibodies to specifically deplete the neutrophils. Depletion with Ly-6G does not have much effect on IFN-γ production. Depletions had no effect on IFN-γ production in response to PMA and ionomycin, demonstrating that the depletion procedures did not incapacitate the cells. Given that IL-12 is essential for the induction of IFN-γ, the reduction of IFN-γ could be due to a depletion of IL-12 producing populations. To exclude the possibility, we found that depletion of various cell types did not significantly abolish the IL-12 in the supernatant in response to stimulations with heat-killed and live bacteria (Fig. 5B).

### Gr-1 expressing populations in BALB/c and C57BL/6 splenocytes

**Figure 6:**
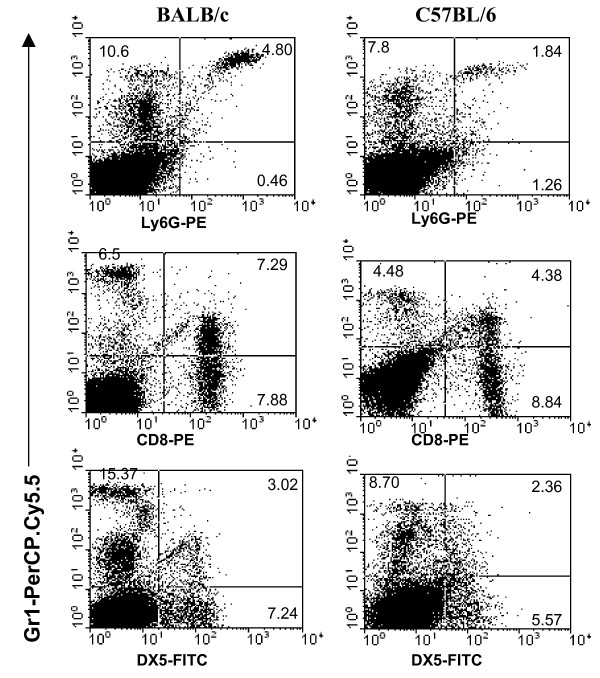
**Identity of Gr-1 expressing cells**. Naïve BALB/c and C57BL/6 splenocytes were stained with various fluorochrome-conjugated antibodies and analyzed by flow cytometry. Quadrant statistics were determined based on staining with appropriate isotype control antibodies. The experiment has been repeated more than 3 times each with different animals to yield similar results.

Since Gr-1^intermediate ^cells contribute to the IFN-γ response, we next determine the identity of these cells in BALB/c and C57BL/6 splenocytes. BALB/c splenocytes generally contained more Gr-1^+ ^cells as well as NK cells compared to C57BL/6 splenocytes (Fig. 6). Besides macrophages and dendritic cells which express Gr-1^intermediate ^[17,18], we found that a higher proportion of BALB/c CD8 T cells (about 50%) and NK cells (about 30%) co-expressed Gr-1^intermediate ^compared to those from C57BL/6. Hardly any CD4 T cells from C57BL/6 and BALB/c express Gr-1^intermediate ^(data not shown).

### Neutrophils make little IFN-γ

**Figure 7:**
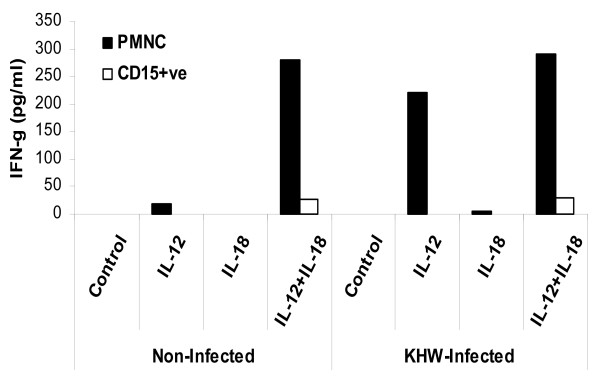
**IFN-γ response of human neutrophils**. Polymorphonuclear cells isolated from human peripheral blood and positively selected CD15^+ ^cells were cultured for 24 h in the presence of IL-12, IL-18 and/or live *B. pseudomallei*. IFN-γ concentrations were determined by ELISA. The experiment had been repeated twice with the same results using cells from different donors.

There had been increasing reports of neutrophils producing IFN-γ upon various stimulations [19-22]. We found that the positively selected Ly-6G^+ ^cells, representing the neutrophils, were only able to make very low amounts of IFN-γ in the presence of IL-12 and bacteria (data not shown), which explains why their depletion did not make much difference to the total IFN-γ produced. We next isolated human neutrophils from peripheral blood to test if they could make IFN-γ. We found that neutrophil preparations varied in their ability to make IFN-γ depending on the presence of contaminating cell-types such as T lymphocytes. When neutrophil preparations were subjected to an additional round of positive selection with CD15 beads, giving rise to a very pure neutrophil population, their ability to make IFN-γ decreased drastically (Fig. 7). This shows that neutrophils are relatively inefficient in making IFN-γ even in the presence of IL-12 and IL-18.

## Discussion

IFN-γ plays a critical role in pathological reactions which could lead to shock and death caused by Gram-negative bacteria or in response to LPS [23,24]. In the murine model of *B. pseudomallei *infection, early hyperproduction of IFN-γ by two days after infection in BALB/c mice is correlated with susceptibility to disease eventually resulting in death [10,11]. To understand the factors contributing to the acute hyperproduction of IFN-γ in response to infection in susceptible BALB/c mice as compared to the relatively resistant C57BL/6 mice, we used naïve splenocytes from both mouse strains to examine their response to *B. pseudomallei*. Our results show that BALB/c splenocytes made more IFN-γ compared to C57BL/6 splenocytes when stimulated with live bacteria, mimicking the IFN-γ hyperproduction by infected BALB/c mice *in vivo*. Although the *in vitro *model is an oversimplification as it excludes cell migration dynamics, it provides a convenient way of investigating inherent factors that contribute to the propensity of BALB/c cells to make IFN-γ in response to live bacteria. However, when stimulated with heat-killed bacteria, this trend is reversed. C57BL/6 and BALB/c mice have generally been classified as high and low IFN-γ responders respectively to account for their innate resistance and susceptibility to pathogens [25,26]. In response to heat-killed bacteria, C57BL/6 splenocytes indeed made more IFN-γ than BALB/c, conforming to this model likely through the significantly higher IL-12 production.

We showed that BALB/c splenocytes were poorer in controlling bacterial infection. This would agree with *in vivo *data suggesting that high bacterial loads lead to hyperproduction of IFN-γ [11,12]. It had been reported that peritoneal cells isolated from C57BL/6 mice were more potent at microbicidal activity against *B. pseudomallei *compared to those from BALB/c mice [27]. However, higher IFN-γ observed in *B. pseudomallei *infected BALB/c than C57BL/6 splenocytes was not due to difference in the cell viability from the two strains of mice, although there could be more subtle damages to cells which the XTT assay failed to detect.

Although IL-12 is absolutely essential for IFN-γ production, we found that the higher IFN-γ production did not result directly from higher IL-12 concentrations in BALB/c when stimulated with live bacteria. In fact, BALB/c cells made more IL-10, abrogation of which increased IFN-γ production. This means that IL-12 concentration is not limiting and differences in IL-10 concentrations between the mouse strains are not big enough to tip the balance in IFN-γ production. IL-10 and IFN-γ are antagonistic to each another [28-30]. IL-10 can inhibit IFN-γ production by NK cells in the presence of antigen presenting cells (APCs) through the inhibition of IL-12 [31,32]. This could explain why we did not see higher IL-12 level in BALB/c mice, and it also implies that co-stimulatory factors other than IL-12 (such as IL-15 and IL-18) were involved in IFN-γ response to *B. pseudomallei *infection [14,16]. Since only live but not dead bacteria induced IL-10, this suggests that *B. pseudomallei *possess active mechanisms to trigger IL-10 production. It is possible that the high IL-10 seen in BALB/c in response to live bacteria could be a consequence of the immune system trying to counteract and suppress the increasing inflammation.

We did not find any obvious differences in the cell types making IFN-γ between BALB/c and C57BL/6 splenocytes. However, BALB/c splenocytes contained more Gr-1^high ^and Gr-1^intermediate ^cells, as well as NK cells. As NK cells are major IFN-γ producers in innate immunity, this translates to more IFN-γ. Although depletion of CD8 T cells did not affect IFN-γ production, CD8 T cells could produce IFN-γ in response to *B. pseudomallei *as examined by the IFN-γ capture assay (data not shown) and as shown by others [14,16]. It is possible that the CD8 depletion from the bulk population also relieves a suppressive effect on the other cells making IFN-γ. The other explanation is they contribute only little in terms of quantity to the total amount so when they were depleted, the remaining population was enriched for the other cells making IFN-γ. In addition, we found that Gr-1^intermediate ^cells contributed to the IFN-γ response. BALB/c splenocytes had higher numbers of Gr-1^intermediate ^expressing CD8 T cells and NK cells compared to C57BL/6 and could explain why BALB/c cells made more IFN-γ. This apparently requires a live bacterial infection because although the same cells made IFN-γ in response to dead bacteria, they did not contribute to a higher response in BALB/c. A live infection allows for various pathogenic mechanisms of the bacteria, including the Type III secretion systems (TTSS), to operate. This may be necessary to allow for continual activation of cells or other factors such as IL-15 which could act as a co-stimulus for NK cells to make IFN-γ [33], and IL-27 which could act as a co-stimulus for T cells [34] to induce a high IFN-γ response. Live bacteria could also induce caspase-1 activation in macrophages, leading to secretion of IL-1β and IL-18 [35]. As BALB/c splenocytes constitutively produced more IL-18 than C57BL/6, and IL-18 is at least partially required for IFN-γ, this could be an additional factor that contributes to the hyperproduction of IFN-γ in BALB/c splenocytes.

Several published studies employ Gr-1 antibodies to deplete neutrophils *in vivo *[36-38]. In our cell depletion studies, anti-Gr-1 depletion significantly reduced IFN-γ production. However, since the anti-Gr-1 antibody recognizes both Ly-6G and Ly-6C markers and are not exclusive for neutrophils, we excluded the involvement of neutrophils through depletion of Ly-6G^+ ^cells. Ly-6G is found almost exclusively on neutrophils and represents the Gr-1^high ^cells. This means that the Gr-1 antibody is depleting other cell-types expressing Ly-6C or the Gr-1^intermediate ^cells. Haque *et al*. had recently shown F4/80 macrophages to be producing IFN-γ during *B. pseudomallei *infection *in vivo *[16]. We did not further characterize the Gr-1^intermediate ^population making IFN-γ, but we did find the expression of Gr-1^intermediate ^on T cells and NK cells, which likely express Ly-6C as Ly-6G is not found on these cells. Others had shown that Ly-6C expressing NK cells [39] and CD8^+ ^CD44^hi ^Gr-1^+ ^T cells [40] make IFN-γ. As BALB/c splenocytes contained more NK cells and Gr-1 expressing NK and T cells, this could be a factor contributing to higher IFN-γ production upon live bacterial encounter. Furthermore, we found that highly purified neutrophils make very little IFN-γ even in the presence of IL-12 and IL-18 and minute numbers of contaminating lymphocytes in neutrophil preparations could contribute to the IFN-γ effect. Thus, it is imperative for studies examining the production of IFN-γ by neutrophils to use highly purified neutrophils and to exclude the contribution of contaminating cell-types. In fact, Schleicher *et al *recently found that minute numbers of contaminating CD8^+ ^T cells or CD11b^+^CD11c^+^NK cells are the source of IFN-γ in IL-12/IL-18-stimulated mouse macrophage populations [41]. This highlights the need for caution in interpreting data due to the possible presence of minute numbers of contaminating but high IFN-γ producing cells.

It has been reported that IL-18 could induce neutrophilia in mice by acting on macrophages to produce IL-6, which in turn causes neutrophilia [42]. Thus, the constitutively high IL-18 in BALB/c mice likely contributes to more neutrophils present in BALB/c splenocytes. Furthermore, addition of IL-18 to splenocytes in culture induced the production of GM-CSF, IFN-γ and IL-6 [42]. As susceptible BALB/c mice show hyper-inflammation upon infection and succumb to acute disease, this seems to parallel patients with acute melioidosis who exhibit hyper-inflammation [7-9,43]. As the most prominent risk factor for melioidosis is diabetes [1,44], our studies in the susceptible mouse strain may have implication on the immune condition of diabetic individuals such as constitutive inflammation [45,46] which predisposes them to acute disease.

## Conclusion

An active infection is required to induce a hyperproduction of IFN-γ in BALB/c compared to C57BL/6 splenocytes. The higher production of TNF-α, IL-1β, IL-6 and IL-18, and the presence of more neutrophils contribute to a more intense inflammation seen in BALB/c splenocytes. The higher intracellular bacterial loads in BALB/c splenocytes likely contribute to more inflammation. Furthermore, the higher numbers of NK cells and Gr-1^intermediate ^expressing cells in BALB/c splenocytes also contribute to more IFN-γ. Future investigations into how each cell population interacts with live bacteria could shed more light on the control of inflammation in the different mouse strains.

## Methods

### Bacterial strains

Virulent *Burkholderia pseudomallei *strain KHW used in this study is a local clinical isolate from a fatal case of melioidosis in Singapore. The isolate was identified as *B. pseudomallei *based on colonial morphology, API20 NE tests (BioMerieux, Marcy I'Etoile, France), and 16S RNA sequence.

### Infection with *B. pseudomallei*

Bacteria were cultured on Trypticase soy agar (TSA) (Difco Laboratories, Detroit, MI) for 24 h at 37°C. Colonies were cultured overnight in LB medium, and a 1:20 dilution prepared and cultured for another 4 h. Bacterial number was estimated spectrophotometrically (1 OD_600 _= 2 × 10^6 ^CFU/μl). Heat-killed or heat-inactivated bacteria were prepared by treatment of overnight bacterial culture at 80°C for 1 h. Dead bacteria were centrifuged at 10,000 g, washed twice with sterile PBS and stored at -20°C.

### Preparation and stimulation of splenocytes *in vitro*

Naïve mice were killed by CO_2 _asphyxiation. Spleens were removed aseptically and single cell suspensions prepared by passing through sterile mesh and erythrocytes were lysed. Cells were placed in antibiotic-free complete antibiotic-free RPMI 1640 medium (Sigma, St. Louis, MO) supplemented with 10% Fetal Bovine Serum (FBS) (Hyclone Laboratories, Logan, UT), 200 mM L-glutamine, 100 units/ml penicillin and 100 μg/ml streptomycin at a concentration of 2 × 10^6 ^cells in 0.5 ml of medium. Cells were stimulated with heat-killed *B. pseudomallei *at a ratio of bacteria to cell of 5:1 or infected with live *B. pseudomallei *at a multiplicity of infection (MOI) of 5:1. Treatment of cells with phorbol myristate acetate (PMA) (50 ng/ml) and ionomycin (1 μg/ml) served as positive control. The following antibodies were used for neutralization studies: anti-IL-12 (polyclonal goat IgG, R&D Systems, Minneapolis, MN), goat IgG isotype control, anti-IL-18 (clone 93–10C, MBL, Nagoya, Japan), anti-IL-10 (clone JES5-2A5, Biolegend, San Diego, CA), and rat IgG1 isotype control (R&D Systems). At 90 minutes after inoculation of bacteria, 250 μg/ml of kanamycin was added into each well to inhibit the growth of the extracellular bacteria. Supernatants were harvested at the indicated time points after treatment and stored at -20°C.

### Cell viability determination

Viability of splenocytes was determined by the XTT assay kit (Roche Diagnostics, Mannheim, Germany) according to manufacturer's instructions. Briefly, 2 × 10^6 ^splenocytes were cultured in 96-well plate at a final volume of 100 μl antibiotic-free culture medium. Infection was performed as described above. Ninety minutes after inoculation of bacteria, 250 μg/ml of kanamycin was added into each well to kill extracellular bacteria. XTT labeling mixture was prepared by adding electron coupling reagent with XTT labeling reagent. 50 μl of labeling reagent were added into each well containing the splenocytes at 4 h before the measurements were recorded. The absorbance of the samples was measured spectrophotometrically using a microplate reader at a wavelength of 490 nm.

### Bacterial load determination

To estimate the total bacteria count *in vitro*, splenocytes were cultured in antibiotic-free medium, and cell were harvested and lysed with 0.1% Triton-X100 at 4 h post-infection. For the intracellular bacteria load, cells were infected for 2 h before kanamycin was added. Cells were harvested and lysed with 0.1% Triton-X100 2 h later. Serial dilutions were made from the suspension and plated onto selective agar plates to obtain the bacterial counts.

### Magnetic cell separation for cell-type purification

Single cell suspensions from spleens of mice were washed with MACS buffer (PBS supplemented with 2 mM EDTA and 1% FBS) and CD4, CD8, NK cells were purified using anti-CD4, anti-CD8, and anti-DX5 microbeads while the Gr-1^+ ^cells were stained with anti-Gr-1 antibodies (RB6-8C5, Biolegend) followed by incubation with streptavidin microbeads (Miltenyi Biotec, Bergisch Gladbach, Germany), neutrophils were stained with Ly-6G-PE followed by anti-PE microbeads before passing through the magnetic columns. All separation procedures were performed according to the manufacturer's guidelines. After incubation for 30 min at 4°C, the positive cells were depleted using MS column or LD column (Miltenyi Biotec), washed and resuspended in antibiotic-free medium.

### Cytokine determination by ELISA

Supernatants were harvested at the indicated time points after treatment and stored at -20°C for ELISA. Mouse IFN-γ, IL-12, IL-10, TNF-α, and human IFN-γ ELISA assay kits were purchased from Bender MedSystem (Vienna, Austria), IL-1β and IL-18 ELISA kits from BD Biosciences, whereas IL-6 ELISA kit was purchased from Biolegend. All ELISAs were performed according to manufacturer's instructions. Cell culture supernatants were diluted 2 fold and assayed in duplicates. The detection limits for these assays were 15.6 pg/ml for IFN-γ, TNF-α, IL-6 and IL-1β, 31.2 pg/ml for IL-12 and IL-18, 39.0 pg/ml for IL-10, and 1.5 pg/ml for human IFN-γ.

### Flow cytometric analysis

Antibodies used for cell surface marker staining were purchased from BD Pharmingen (San Diego, CA) unless otherwise stated: anti-CD8-PE (53-6.7, rat IgG2a), anti-CD4-biotin (GK1.5, rat IgG2b), anti-CD8-biotin (53-6.7, rat IgG2a), anti-CD3-FITC (145-2C11, Hamster IgG1) anti-Ly-6G-PE (1A8, rat IgG2a), anti-Gr-1-PerCP-Cy5.5 (RB6-8C5, rat IgG2b), anti-pan NK-FITC (DX5, rat IgM, Biolegend). Splenocytes were adjusted to 1 × 10^6 ^cells and stained with cell surface specific antibodies or isotype control antibodies for 30 min on ice. Where the biotin conjugated antibodies were used, staining with SAV-PE/SAV-FITC or secondary antibody was performed for 15 min at 4°C. After washing, the cells were analyzed by FACScan (Becton Dickinson, San Jose, CA) flow cytometer.

### Interferon γ capture assay

Secretion of IFN-γ was analysed with an IFN-secretion assay kit (Miltenyi Biotec) according to the manufacturer's instructions. Briefly, infected splenocytes were cultured for 16 h at 37°C, 5% CO_2 _in medium. At the conclusion of the assay, cells were incubated with optimal concentration of antibodies for surface marker staining together with PE-conjugated anti-IFN-γ (Miltenyi Biotec) for 30 min at 4°C. Cells were than washed and analyzed by flow cytometry. Controls included isotype-matched control antibodies conjugated to identical fluorochromes.

### Isolation of human neutrophils from peripheral blood

Human neutrophils were isolated from healthy adult blood donors (National University Hospital Blood Donation Centre). Briefly the buffy coats were diluted 2-fold in PBS and centrifuged through a Ficoll-Paque Plus (Amersham Pharmacia Biotech, Uppsala, Sweden) gradient to separate the erythrocytes/neutrophils from peripheral blood mononuclear cells (PMBC). The plasma, PBMC, and the Ficoll layers were removed by Pasteur pipette, and the erythrocytes/PMNC pellets were transferred into new tubes. Erythrocytes were lysed by osmotic shock using 0.2% NaCl solution and an equal volume of 1.6% NaCl were then added to reconstitute to a balance salt solution containing 0.9% NaCl. To remove minute contaminating lymphocytes, monocytes and NK cells, neutrophils were purified by positive selection with anti-CD15 microbeads (Miltenyi Biotec) according to manufacturer's instructions. Crude PMNC and CD15 positively selected cells were seeded at 2 × 10^6 ^cells per well in 0.5 ml of medium and stimulated with live or heat-killed *B. pseudomallei *in the presence or absence of human IL-12, IL-18 or both. Untreated cells or uninfected cells treated with cytokines serve as controls. Supernatant were harvested at 24 h for ELISA.

### Statistical analyses

Statistical significance was determined by Student's t-test. A *p *value of < 0.05 is regarded as statistically significant.

## Authors' contributions

YHG is responsible for conceiving, directing the study and writing of the manuscript. GCK carried out the experiments and contributed to the design of experiments and drafting of part of the manuscript. Both authors read and approved the final manuscript.
